# The Benefits and Risks of Prophylactic Central Neck Dissection for Papillary Thyroid Carcinoma: Prospective Cohort Study

**DOI:** 10.1155/2015/571480

**Published:** 2015-07-13

**Authors:** Doh Young Lee, Kyoung Ho Oh, Jae-Gu Cho, Soon-Young Kwon, Jeong-Soo Woo, Seung-Kuk Baek, Kwang-Yoon Jung

**Affiliations:** Department of Otorhinolaryngology-Head and Neck Surgery, Korea University College of Medicine, Seoul 136-705, Republic of Korea

## Abstract

*Objectives*. This study evaluated the benefits of performing prophylactic central neck dissection (CND) with total thyroidectomy (TT) in management of papillary thyroid carcinoma (PTC) patients who were clinically node-negative at presentation.* Methods.* A total of 257 patients with stage T1 or T2 PTC and without preoperative evidence of lymph node involvement (N0) were enrolled in this prospective study. The patients were randomly assigned to two groups: (1) a total thyroidectomy (TT) group (*n* = 104) or (2) a TT plus CND group (*n* = 153). The two groups were compared for their perioperative data, complication rates, disease recurrence rates, and clinical outcomes.* Results*. The two groups of patients were similar in age, sex ratio, follow-up duration, and tumor size (*P* = 0.227, 0.359, 0.214, and 0.878, resp.). The two groups showed similar rates of disease recurrence (3.9% in the TT group versus 3.3% in the TT plus CND group); however, complications occurred more frequently in the TT plus CND group; especially transient hypocalcemia (*P* = 0.043).* Conclusions.* Patients treated with TT plus CND had a higher rate of complications with similar recurrence rate. We believe that CND may not be routinely recommended when treating patients with PTC.

## 1. Introduction

While papillary thyroid carcinoma (PTC) accounts for 80–85% of all thyroid malignancies, the 10-year survival among PTC patients who receive appropriate treatment is >90% [1]. However, in 40~90% of PTC cases, cervical lymph node metastases are detected at the time of initial diagnosis or recurrence [2, 3]. Although PTC recurrence in the cervical neck region adversely affects the patient's quality of life and mandates additional treatment, the impact of such lymph node metastases on patient survival remains a subject of debate.

The role of prophylactic central compartment node dissection (CND) performed during the initial surgery also remains controversial [4–6]. Given the excellent overall survival rates associated with PTC and the potential morbidity associated with reoperative cervical surgery, prophylactic CND may seem to be appropriate, as it may decrease the disease recurrence rate by extirpating level VI lymph nodes [7]. However recently, decisions regarding the extent of thyroidectomy and neck dissection to be performed have tended to be made by focusing on rates of CND-associated complications, and reducing the incidence of postsurgical complications is often prioritized if the patient survival rates with and without CND are similar [8]. Therefore, the role of prophylactic CND should be reevaluated by considering both the oncologic outcomes and complication rates.

Several studies have evaluated the effects of prophylactic CND when performed in treating PTC [9–13] and the majority found no significant difference in the rates of disease recurrence or long-term complications between patients having undergone a total thyroidectomy (TT) or a TT plus prophylactic CND. However, there was a trend toward disease lower recurrence rates among patients who received TT plus CND, especially when the combined procedure was performed by a high-volume surgeon. We conducted this prospective randomized cohort study to evaluate the relative benefits of performing routine TT or TT plus prophylactic CND in treatment of patients with PTC. Our findings address some weaknesses in previous studies which used a retrospective analysis and also show the clinical results which can be achieved by a single high-volume surgeon.

## 2. Materials and Methods

### 2.1. Study Population

From January 2006 to December 2010, this study enrolled a total of 257 patients (104 in a TT group and 153 in a TT plus CND group). The inclusion criteria for enrollment were as follows: (1) having undergone a total thyroidectomy; (2) preoperative diagnosis of PTC by ultrasonography guided fine-needle aspiration cytology; (3) clinical stage T1 or T2 PTC without gross extrathyroidal extension; (4) no clinical evidence of neck node involvement (central or lateral); (5) patients aged ≥20 years. The enrollment exclusion criteria were as follows: (1) a diagnosis other than PTC; (2) patients with a mixed tumor; (3) patients with a thyroidectomy; (4) patients with inadequate follow-up data; (5) patients with preoperative evidence of nodal disease or who had undergone lymph node dissection of any lateral compartment during the initial operative intervention. If gross findings during surgery revealed an obvious extrathyroidal extension of the tumor, the patient was excluded from enrollment and underwent a routine central neck dissection. The data gathered for each patient included demographic information, histopathologic characteristics of the primary tumor, and lymph node pathology results. Postoperative complications were recorded with particular emphases on the incidence of RLN palsy, rates of postoperative bleeding or seroma, and rates of temporary and permanent hypoparathyroidism.

### 2.2. Study Protocol

The study protocol was approved by the Institutional Review Board (IRB) of the Korea University College of Medicine. All study patients underwent a total thyroidectomy and were randomly assigned to the TT group (*n* = 104) or TT plus CND group (*n* = 153). No patient in either group received a drainage tube, and each patient was routinely discharged two days after surgery if there was no significant complication which required treatment in the hospital. All surgeries were performed by one surgeon (KY Jung).

Each patient underwent a preoperative evaluation which included a fine-needle biopsy of the primary tumor and a determination of lymph node status by both a physical examination and dedicated neck ultrasonography. Vocal fold movement was assessed during routine preoperative and postoperative laryngoscopic examinations. Temporary vocal cord palsy was defined as decreased or absent vocal cord mobility which resolved within 6 months after surgery. Permanent vocal cord palsy was defined as vocal cord dysfunction persisting > 6 months after the initial surgery. Serum calcium levels were obtained at the following time points: preoperatively and then twice a day (6:00 AM, 4:00 PM) on postoperative days (PODs) until discharge. Symptomatic hypocalcemia (such as perioral and distal digital paresthesias, tetany, and palpitation) was regarded as being present if the calcium level was lower than 8 mg/dL and patient complained of any of the above symptoms, irrespective of the duration of their hospital stay. Routine calcium supplementation was given to all patients with total thyroidectomy as follows (fixed dose per day): CaCO_3_ 1,000 mg three times a day with 0.25 *μ*g calcitriol twice a day. Permanent hypocalcemia or hypoparathyroidism was defined as an ongoing need for calcium or vitamin D supplementation that lasted > 12 months. Radioactive iodine (RI) remnant ablation was performed after surgery with iodine-131 according to the American Thyroid Association Guidelines [14]. RI therapy was provided after thyroid hormone withdrawal or stimulation with recombinant thyroid stimulating hormone (thyrotropin alfa, Thyrogen; Genzyme, Inc., Framingham, MA, USA).

Patients were routinely followed up every 3 months during the first postoperative year and on a yearly basis thereafter. During each follow-up visit, the patient received a routine physical examination which included neck ultrasonography and assays for thyroglobulin and thyroglobulin antibody levels. Additionally, each patient received an annual whole body scan. Routine physical examination was performed during the follow-up, and ultrasonography guided fine-needle aspiration was performed whenever there was a palpable neck lymph node. If the suppressed Tg level was over 2 ng/mL and whole body scan revealed increased uptake, ultrasonography was also performed to detect the recurrent disease. Small nonpalpable lesion (less than 8 mm in ultrasonography) is initially treated with RI therapy if it is in the tumor bed or level VI area. If lateral neck was involved, compartmental neck dissection following RI therapy was performed regardless of the size of the lesion.

### 2.3. Statistical Analysis

Results for continuous variables are presented as the mean value ± standard deviation (SD), and results for categorical variables are presented as frequencies and group percentages. The independent* t*-test and chi-square test were used to compare results for continuous and categorical values, respectively. Cumulative disease recurrence rates were calculated using a life table, and differences in patient outcomes were analyzed using the log-rank test. All statistical analyses were performed using IBM SPSS Statistics for Windows, Version 20.0. Armonk, NY: IBM Corp. *P* values < 0.05 were considered statistically significant.

## 3. Results

This study enrolled a total of 257 PTC patients who were randomly assigned to two different groups (104 in the TT group and 153 in the TT plus CND group). The two groups were similar regarding patient age, sex ratio, follow-up duration, and tumor size (*P* = 0.227, 0.359, 0.214, and 0.878, resp.) (Table 1). Additionally, the two groups of patients had similar operation times. Demographic characteristics of the patients in this study are summarized in Table 2.

Postoperative pathology results showed no significant difference in the T stage of patients in each group (*P* = 0.817). Thirteen patients (12.5%) in the TT group were later diagnosed with T3 based on the presence of microscopic extrathyroidal extensions, while 19 patients (13.7%) in the TT plus CND group were recategorized as having T3 (*P* = 0.432). Although parathyroid tissue was inadvertently excised during both the TT and TT plus CND procedures (*P* = 0.437), parathyroid autotransplantation was performed more often in the TT plus CND group (*P* = 0.023). Seventy-four patients (71.2%) in the TT group and 112 patients (73.2%) in the TT plus CND group received postoperative RI therapy; however, this difference between groups was not statistically significant (*P* = 0.405).

**Figure 1:**
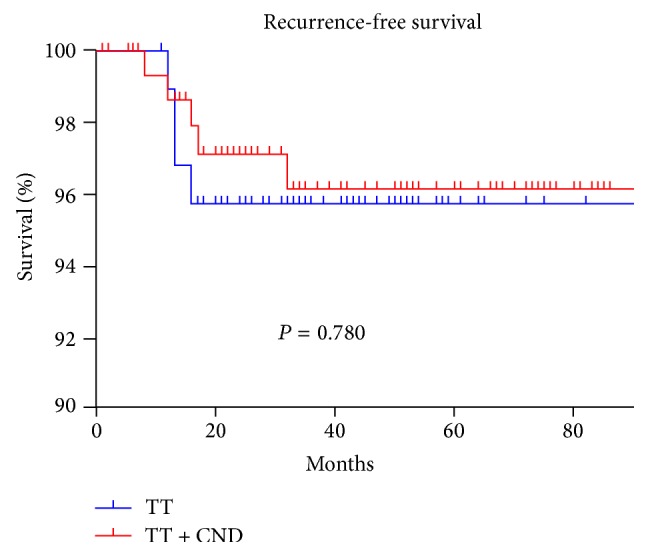
Recurrence rate according to the operation type.

There was no significant difference in the disease recurrence rates in the two groups throughout the study period (3.9% in the TT group versus 3.3% in the TT plus CND group) (Figure 1). However, the complication rate in the TT plus CND group was significantly higher than that in the TT group, especially the occurrence of transient hypocalcemia (*P* = 0.043). Additionally, there were higher incidences of transient or permanent vocal cord paralysis, postoperative bleeding, and seroma, in the TT plus CND group; however, these differences were not statistically significant. No deaths occurred in either study group. The mean disease-free durations were 13.2 months in the TT group and 14.2 months in the TT plus CND group. There were no significant differences between groups regarding the site of disease recurrence, the type of operation performed, or the mean number of nodes and positive nodes at the time of reoperation (Table 3).

## 4. Discussion

There is a general consensus that either CND or another type of neck dissection is indicated when tumor-positive lymph nodes are detected by ultrasonography or palpation [14–17]. The current American Thyroid Association Guidelines recommend that CND may be performed in patients with papillary thyroid carcinoma with clinically uninvolved central neck lymph nodes, especially for advanced primary tumors (T3 or T4) [14]. For patients with small (T1 or T2), noninvasive, clinically node-negative PTCs and most follicular cancers, prophylactic CND may not be appropriate according to the guidelines. However, the use of elective prophylactic CLN remains controversial when preoperative examinations do not suggest lymph node involvement [14]. Residual metastatic lymph nodes represent the most common cause of tumor recurrence [18], and some investigators believe that CND reduces the risk of recurrence and improves patient survival rates [19]. In contrast, other investigators insist that total thyroidectomy and RI ablation therapy without CLN constitute sufficient treatment when a tumor-positive lymph node is not detected by ultrasonography or palpation.

To the best of our knowledge, ours is the first prospective randomized cohort study to evaluate the oncologic and surgical outcomes of Asian patients treated using prophylactic CND, and the results suggest that prophylactic CND may not be necessary when treating early T1/T2 stage, N0 thyroid cancer. Even when performed by a high-volume surgeon, prophylactic CND failed to decrease the recurrence rate of T1/T2 thyroid cancer compared to the recurrence rate seen after TT alone. Furthermore, the complication rate associated with CND plus TT was significantly higher than that associated with TT alone. There was no difference in short-term recurrence in the CND group but there was higher temporary hypocalcemia, partially due to inadvertent removal of a parathyroid gland. Therefore, we suggest that prophylactic CND should not be recommended when treating N0 category PTC patients who do not show evidence of an extrathyroidal tumor extension during a preoperative evaluation.

Some studies have reported lower postoperative serum thyroglobulin levels and reduced rates of disease recurrence in the central neck region, while other studies have reported no difference in PTC recurrence and even upstaging of disease and subsequent overuse of adjuvant radioactive iodine therapy following detection of central neck lymphadenopathy [2, 4–6, 20, 21]. A previous report concerning the feasibility of conducting a prospective randomized controlled trial to examine the benefits received by performing prophylactic CND showed that several thousand patients would be required to attain a sample power sufficient to detect a difference in rates of complications and disease recurrence [22]. Given the prohibitively large sample size required, such a study has not been deemed feasible to perform.

Several meta-analyses and a systematic review have been conducted in attempts to overcome this problem, and a recent meta-analysis suggested that patients who receive prophylactic CND may have a lower risk of disease recurrence compared to those who do not undergo CND [10]. In that meta-analysis with six studies, a 7.9% recurrence rate was found in a TT group compared to 4.7% in a TT plus prophylactic CND group, for a relative risk of 0.59; however this difference was not statistically significant. Moreover, results of another meta-analysis suggested that prophylactic CND was not beneficial, by showing overall disease recurrence rates of 2.02% in a prophylactic CND group and 3.92% in a TT alone group [23]. Additionally, the two groups showed similar recurrence rates in the central and lateral neck regions. Shan et al. reported that, compared with use of TT plus CND, use of TT alone resulted in less surgical morbidity in the immediate postoperative period and an identical locoregional disease recurrence rate [24]. These previous meta-analyses provided high quality evidence regarding the benefits of prophylactic CND and included information concerning each study's inclusion and exclusion criteria, patient age range, extent of thyroidectomy, and range of tumor sizes.

The results of our current study are in accordance with those in the previous meta-analyses, in that treatment with TT plus CND resulted in higher rates of postoperative complications compared with the complication rates seen when using TT alone. Additionally, this higher rate of complications also occurred when TT plus CND was performed by a high-volume thyroid surgeon. Sosa et al. [25] were the first investigators to report a significant association between surgical volume and patient outcomes following thyroidectomy, and several subsequent reports have confirmed that a surgeon's experience and volume are significantly correlated with surgical outcomes [26–32]. Our study also revealed a significant correlation between surgical extent and increased complications following thyroidectomy, even when the operation was performed by a high-volume surgeon. Hauch et al. [33] reported overall complication rates of 7.6% and 14.5%, for lobectomy and TT, respectively. While previous studies used disease codes to identify complications, we identified complications by reviewing patient medical charts. Thus our data collection process may have yielded more accurate results and been the reason for the higher complication rates reported in our study. The positive correlation between surgical extent and complication rates supports recent trends that seek to minimize the extent of surgery performed when treating patients with PTC.

Our study has several limitations that should be mentioned. First, this study did not include a patient voice evaluation or quality of life assessment, and both of these factors can affect a patient's overall condition nearly as much as postoperative complications [34]. Results for patient voice quality and quality of life may have altered the results of our study. Second, the relatively short follow-up time used in our study may have biased the results. Patients treated for PTC have a relatively long survival time, and we were unable to evaluate the long-term outcomes of patients who received prophylactic CND. If it were available, long-term disease recurrence and survival data might have also affected our final results. Third, due to the small cohort size and short follow-up time in our current study, we were unable to perform subgroup analyses to examine whether any patient subgroup may have benefitted from treatment with prophylactic CND. Moreover, as Carling et al. reported [22], a considerable number of patients are needed to clarify the role of CND by prospective study and our study is significantly underpowered. Fourth, there was an imbalance of the number of patients in each group, which was due to exclusion by inadequate follow-up. A further study with larger numbers of patients and longer follow-up times could be conducted to address the limitations of our current study.

Despite its limitations, our study has value by being the first prospective randomized study to evaluate the benefits of prophylactic CND in treatment of PTC with T1/T2. Considering that the aforementioned large cohort study could not be performed at a single medical center, our results provide a rationale for conducting both a further prospective multicenter study and a meta-analysis of prospective studies. Unless proven otherwise by a further prospective study, we believe that it may be unnecessary to perform prophylactic CND when treating patients with PTC with N0 at presentation.

## Figures and Tables

**Table 1:** Demographic and perioperative data.

	TT (*n* = 104)	TT plus CND (*n* = 153)	*P* value
Age	51.6 ± 3.4	52.3 ± 2.3	0.227

Sex ratio (M : F)	1 : 5.5	1 : 4.1	0.359

Follow-up duration (months)	49.2 ± 15.9	55.2 ± 11.3	0.214

Tumor size (cm^3^)	1.6 ± 1.4	1.7 ± 1.2	0.878

Operation time (min)	93.2 ± 22.4	95.6 ± 17.9	0.093

T stage			0.817
pT1	59 (56.7%)	83 (52.2%)	
pT2	18 (17.4%)	29 (18.9%)	
pT3	27 (25.9%)	41 (26.9%)	

N stage			—
pN0	NA^*∗*^	118 (77.2%)	
pN1a	NA	35 (22.8%)	

Multiplicity	34 (32.7%)	58 (37.9%)	0.497

Parathyroid inadvertently excised^†^	6 (5.8%)	11 (7.2%)	0.437
Parathyroid autotransplantation^‡^	8 (7.7%)	22 (14.4%)	0.033

Postoperative RAI	74 (71.2%)	112 (73.2%)	0.405

^*∗*^Perithyroidal lymph node was incidentally harvested in 13 patients (12.5%), all of which were negative for malignancy; ^†^detected in pathological review, not during the surgery; ^‡^devascularized parathyroid during the surgery.

**Table 2:** Comparison of complication rates.

	TT	TT plus CND	*P* value
Vocal cord paralysis			
Transient	2 (1.9%)	5 (3.3%)	0.245
Permanent	0	2 (1.3%)	0.211

Hypoparathyroidism			
Transient	21 (20.3%)	56 (36.6%)	0.043
Permanent	2 (1.9%)	5 (3.3%)	0.245

Bleeding	1 (0.9%)	2 (1.3%)	0.705

Seroma	2 (1.9%)	3 (2.0%)	0.817

**Table 3:** Characteristics of the patients with recurrence.

Number	Group	pT	RAI	Recurrence-free duration (months)	Recurrence site	Reoperation	Number of nodes at reoperation	Number of positive nodes
1	TT	2	150	12	Neck	CND + MRND	14	3
2	TT	1	30	13	Neck	SND	8	1
3	TT	1	30	16	Tumor bed	CND	6	1
4	TT	1	30	13	Tumor bed, neck	CND + MRND	26	4
5	TT plus CND	1	30	12	Neck	SND	11	2
6	TT plus CND	2	150	8	Tumor bed, neck	CND + MRND	19	3
7	TT plus CND	1	100	17	Tumor bed, neck	CND + MRND	37	5
8	TT plus CND	3	150	16	Tumor bed, neck	CND + LND	13	3
9	TT plus CND	3	150	32	Neck	LND	17	2
